# Advancing zoo animal welfare through data science: scaling up continuous improvement efforts

**DOI:** 10.3389/fvets.2024.1313182

**Published:** 2024-01-17

**Authors:** Matyas Liptovszky

**Affiliations:** ^1^Perth Zoo, South Perth, WA, Australia; ^2^School of Veterinary Medicine and Science, University of Nottingham, Sutton Bonington, United Kingdom

**Keywords:** animal welfare, zoo, data science, continuous improvement, evidence-based decision making, artificial intelligence, machine learning

## 1 Introduction

Advancing animal welfare in zoos is a multifaceted endeavor that lies at the core of their conservation and educational missions. While zoos have made significant strides in improving the wellbeing of their animal residents, challenges persist in ensuring a comprehensive, evidence-based approach to continuous improvement. Some of these challenges stem from the exponential growth of animal welfare in the last few decades, making it difficult to keep up with ever evolving theories and practical assessment methodologies (1). Others are related to the inherent resource limitations of any organization, including staff time and financial resources dedicated to animal welfare. A significant limitation with the latter is staff time allocated to animal observation and other data collection.

Untapped potential currently exists for data science as a transformative tool to overcome some of these challenges and further advance animal welfare practices in zoos. Data science is an interdisciplinary field that involves the extraction of valuable insights and knowledge from large and complex datasets through a combination of statistical analysis, machine learning, and domain expertise. It encompasses various processes, including data collection, cleaning, and transformation, as well as the application of algorithms and machine learning models to uncover patterns, trends, and correlations within the data. Data science leverages programming languages and tools to process and visualize data, enabling data scientists to interpret and communicate findings effectively. It empowers organizations to make informed decisions, predict future trends, and solve complex problems across diverse domains, from business and healthcare to environmental sciences and beyond. As data continues to proliferate in the digital age, data science plays a crucial role in extracting meaningful knowledge and unlocking the potential for innovation and advancements in numerous fields (2).

The data science lifecycle starts with problem identification and business understanding: the need to deeply understand the operational environment where the data collection and analysis (and any subsequent predictions) will be carried out, and the reasons why we would do that. This provides opportunities for zoos to consider various approaches to how data science can be implemented within existing systems. This can be simple, by analyzing single data sources, or complex, aiming at comprehensive welfare assessments based on all available evidence. A key to the success of this step is domain expertise, in our case expertise from animal care, veterinary and animal behavior and welfare staff. Emphasizing this key first step is also likely an important solution to reduce resistance toward novel technologies and methodologies, as it ensures that the inherent knowledge and expertise of the zoo team are captured.

By adopting a data-driven approach, zoos can capitalize on the wealth of information already available from diverse sources, including readily available daily keeper reports, animal health records, behavioral observations, enclosure data, as well as through emerging technologies such as CCTV footage, environmental sensors, and acoustic recordings. Harnessing the power of data science could allow for efficient and in-depth assessments of animal welfare, enabling zoos to extend their focus to a larger number of animals and make evidence-based decisions for their care. This shift toward data-driven decision-making not only optimizes resource allocation but also leads to a more substantial and positive impact on animal welfare.

Furthermore, integrating data science into daily operations and research can foster collaboration between experts from various fields, including data scientists, academics, and zoo professionals. By encouraging the exchange of knowledge and expertise, this collaborative effort will help zoos address common challenges. Embracing technological change and fostering collaboration will lead to an evidence-based approach for the continuous improvement of animal welfare in zoos. Ultimately, this approach not only benefits the animals under our care but also empowers staff working toward increasingly better animal welfare outcomes and provides vital proof of these efforts for visitors and the broader society.

## 2 Scalability, efficiency, and impact

Efficiently assessing the welfare of animals in zoos is critical to ensure that resources are allocated appropriately and that continuous improvement efforts are most impactful. Many zoos conduct welfare assessments, carried out through careful observation of animals and/or evaluation of data collected by trained staff. This can be, however, labor intensive, limiting the scale and frequency of such evaluations. The need for scalability and efficiency calls for innovative solutions that can harness the vast amount of data collected daily in zoo environments. Zookeepers record data on animal behavior, nutrition, provision of various aspects of care, including environmental enrichment activities, as well as environmental parameters in general, creating valuable datasets for analysis. Veterinarians and other animal health staff likewise record a plethora of health-related data, including clinical signs, diagnostic test results, preventative health measures, anesthesia data, and drug usage.

For over 1,300 zoos the Species360 Zoological Information Management System (ZIMS) serves as a central repository for animal data, and it currently includes millions of husbandry and health records representing over 50 years from over 10 million individuals of 22,000 species (3). By tapping into this wealth of data and embracing data science techniques and technology, zoos can gain valuable insights into animal behavior and health, identify potential welfare issues, and assess the effectiveness of enrichment programs.

Zoos also increasingly generate data in various other ways, including through CCTV and other audiovisual monitoring, or environmental monitoring and control systems. Some of these systems are installed primarily for animal care purposes, but many others exist for security, environmental, or facility management purposes. Data are also collected on a wide range of other aspects of zoo operations, from visitor attendance, demographics, and spending to weather patterns to maintenance records. In most instances, these data sources currently remain untapped from an animal welfare perspective, as frequently these are collated in isolated business systems specific to their core task. The combination of traditional animal care staff-collected data and these novel data sources could foster a more holistic approach to animal welfare assessment, leading to comprehensive and data-driven decision-making, as well as significantly improved effectiveness.

Machine learning algorithms can analyze this wealth of data and identify patterns that may not be immediately apparent to staff. By automating data collection and analysis, zoos can free up resources that were previously spent on these time-consuming tasks, allowing staff to focus on data interpretation, evidence-based decision-making, and finding the most impactful improvement opportunities. This shift toward data-driven decision-making can lead to more targeted and impactful interventions, promoting the wellbeing of a larger number of animals in the zoo's care, while empowering zoo experts to utilize the best available evidence.

While data science in zoos is still in its early stages, there are some examples of narrow-scale successful implementations. Some zoos have already begun using CCTV footage to analyze animal behavior and the use of enrichment activities. Machine learning algorithms can process vast amounts of video data to identify behavioral patterns and evaluate the effectiveness of enrichment strategies (4–7). However, Zuerl at al. reviewed currently available commercial systems, as well as previously published frameworks to automate the analysis of video streams. This study concluded that while the potential is great to utilize machine learning for this purpose, currently there are few frameworks which would be fully suitable for zoos and further development in this fields is needed (4). Researchers have harnessed large-scale ZIMS datasets for various purposes, including understanding cancer risk in mammals, senescence in testudines, and sex differences in survival and aging in wild mammals (8–10). A broader review of technology to monitor zoo animal welfare highlighted that by combining multiple data sources, continuous automated monitoring of affective state could be achieved in real or near-real time. In addition, this could also increase our knowledge of certain, currently not well-understood conditions (11). Regional zoo associations also identified taxon and veterinary advisors to help assessing population level trends, and making recommendations to improve health, nutrition, and holistic animal welfare.

These examples demonstrate the potential of data-driven approaches to improve animal welfare by providing insights into animal behavior and preferences, as well as other biological phenomena. These technologies also allow individual-level analysis, which can provide optimized care practices and reduce redundant activities, for example, the provision of environmental enrichment that has no utility. Overall, this can lead to increasing efficiency, which, in turn, leads to better scalability and overall impact.

## 3 Addressing challenges in implementation

Despite the vast potential of data science, several challenges hinder its widespread implementation in zoo settings. Limited access to relevant knowledge and technology, a lack of understanding of data science principles among zoo professionals, resistance to change, concerns about automating a task currently highly trained staff are entrusted to carry out, incomplete record keeping, and reluctance to share data might be among the common barriers. To overcome these challenges, fostering collaboration between data scientists, academics, and zoo professionals is a key aspect. This collaborative effort can facilitate knowledge exchange and skills development, empowering zoo staff to embrace data-driven approaches confidently. Additionally, investing in training and resources for zoo professionals in data science methodologies can help bridge the gap and facilitate the integration of data science into daily zoo operations, as well as reducing fear and suspicion. Emphasizing the practical applications and benefits of data science in enhancing animal welfare can foster a culture of openness to technological change within zoos.

Ensuring ethical data collection and use practices and addressing potential bias in AI systems are also critical considerations. Respecting human privacy and confidentiality is paramount in data collection and analysis, and challenges around this using automated recording systems, like camera traps, have been previously highlighted in wildlife conservation (12). Zoos must establish robust data privacy and security protocols to protect sensitive information collected either for the primary purpose of improving animal welfare or as a side effect. Moreover, AI systems used for welfare assessments must be carefully designed to avoid bias and ensure fair and accurate evaluations. By integrating ethical considerations into the data science process, zoos can build trust with the public and other stakeholders, including staff.

## 4 Integrating data science into zoo animal welfare

Data science offers opportunities for improving day-to-day zoo operations. By analyzing data on animal behavior, health, and environmental conditions, zoos can optimize resource allocation, develop targeted animal care, veterinary management, and enrichment strategies, therefore enhancing animal welfare. The integration of data science into daily operations fosters a more holistic and data-driven approach to animal care, ultimately improving the quality of life for animals in captivity.

Modern businesses have successfully leveraged data science to optimize processes, gain insights, and make informed decisions, and zoos can benefit from these comparable use cases. Data science is likely already integrated into certain business systems within zoos, and extending this usage to animal welfare can unlock significant benefits. Embracing technological change will position zoos at the forefront of animal welfare innovation and enable them to leverage the full potential of data science for continuous improvement. It also has the potential to ensure the ongoing existence of a social license to operate in an environment that is increasingly critical about industries where live animals are a key part of the business.

The successful implementation of data science in zoos necessitates collaboration among various stakeholders. Data scientists, academics, and zoo professionals each bring unique expertise and perspectives to the table. Collaboration facilitates knowledge exchange, helps address challenges, and encourages the adoption of data-driven approaches. Data scientists can provide guidance on data collection, analysis, and AI model development, while zoo professionals contribute essential insights into animal behavior, care, and welfare priorities. Together, these collaborative efforts ensure that data science serves as a transformative tool for advancing animal welfare in zoos.

## 5 Conclusion

Data science presents a transformative opportunity for advancing animal welfare in zoos. By adopting a data-driven approach, zoos can efficiently and effectively assess the wellbeing of a larger number of animals, optimize resource allocation, and make evidence-based decisions. The integration of diverse data sources allows for comprehensive welfare assessments and enriches our understanding of animal behavior and health. Addressing challenges related to knowledge, technology, and resistance to change requires collaboration and investment in training and resources. Ensuring ethical data collection and use, as well as addressing bias in AI systems are essential considerations. Embracing technological change and fostering collaboration between experts are pivotal for success. Data science offers a compelling path forward, empowering zoos to embrace a more evidence-based, data-driven approach to continuously improve animal welfare. This collective effort could positively impact animals, visitors, and staff alike, further elevating the vital role zoos play in conservation, education, and research.

## Author contributions

ML: Conceptualization, Writing – original draft, Writing – review & editing.
